# Long-term outcome among men with conservatively treated localised prostate cancer

**DOI:** 10.1038/sj.bjc.6603411

**Published:** 2006-10-31

**Authors:** J Cuzick, G Fisher, M W Kattan, D Berney, T Oliver, C S Foster, H Møller, V Reuter, P Fearn, J Eastham, P Scardino

**Affiliations:** 1 Cancer Research UK Centre for Epidemiology, Mathematics and Statistics, Wolfson Institute of Preventive Medicine, St Bartholomew's Medical School, Queen Mary, University of London, Charterhouse Square, London EC1M 6BQ, UK; 2 Department of Quantitative Health Sciences, The Cleveland Clinic Foundation, Cleveland, OH 44195, USA; 3 Department of Histopathology, St Bartholomew's Hospital, London E1 2ES, UK; 4 Department of Medical Oncology, St Bartholomew's Hospital, London EC1A 7BE, UK; 5 Department of Cellular Pathology and Molecular Genetics, Liverpool University Hospital, Liverpool L1 3GA, UK; 6 King's College, Thames Cancer Registry, London SE1 3QD, UK; 7 Department of Pathology, Memorial Sloan Kettering Cancer Center, New York, NY 10021, USA; 8 Department of Urology, Memorial Sloan-Kettering Cancer Center, 1275 York Avenue, New York, NY 10021, USA

**Keywords:** localised prostate cancer, prognostic factors

## Abstract

Optimal management of clinically localised prostate cancer presents unique challenges, because of its highly variable and often indolent natural history. There is an urgent need to predict more accurately its natural history, in order to avoid unnecessary treatment. Medical records of men diagnosed with clinically localised prostate cancer, in the UK, between 1990 and 1996 were reviewed to identify those who were conservatively treated, under age 76 years at the time of pathological diagnosis and had a baseline prostate-specific antigen (PSA) measurement. Diagnostic biopsy specimens were centrally reviewed to assign primary and secondary Gleason grades. The primary end point was death from prostate cancer and multivariate models were constructed to determine its best predictors. A total of 2333 eligible patients were identified. The most important prognostic factors were Gleason score and baseline PSA level. These factors were largely independent and together, contributed substantially more predictive power than either one alone. Clinical stage and extent of disease determined, either from needle biopsy or transurethral resection of the prostate (TURP) chips, provided some additional prognostic information. In conclusion, a model using Gleason score and PSA level identified three subgroups comprising 17, 50, and 33% of the cohort with a 10-year prostate cancer specific mortality of <10, 10–30, and >30%, respectively. This classification is a substantial improvement on previous ones using only Gleason score, but better markers are needed to predict survival more accurately in the intermediate group of patients.

The natural history of prostate cancer is highly variable and difficult to predict. Over treatment of asymptomatic patients is a serious problem leading to substantial morbidity. Introduction of prostate-specific antigen (PSA) testing in some countries has exacerbated this problem, leading to a much higher reported incidence rate, but having little influence on mortality rates (Evans and Møller, 2003). In the United States, where PSA testing has been common, the incidence to mortality ratio is about 7.6. Conversely, in the United Kingdom, where PSA testing is not performed routinely, the ratio is approximately 2.5. Autopsy series have confirmed that histologically proven prostate cancer can be identified in approximately 40% of men over 50 years of age who die of other causes (Breslow *et al*, 1977; Sakr *et al*, 1994). This is about four times higher than the lifetime risk for American men to be diagnosed with prostate cancer (approximately 11%), indicating that more intensive screening is likely to uncover even more indolent disease. This had led many countries, in particular the United Kingdom, to endorse a more conservative approach to disease detection and management. In the UK, PSA screening is not endorsed by the health service (although it is available on request) and radical prostatectomy or radiation therapy is not common practice. However, this approach is not without problems, since a substantial proportion of men develop progressive disease from which they ultimately die. Furthermore, conservative management can lead to considerable anxiety, especially when the clinical outcome is so uncertain.

Several studies have attempted to document the long-term risk of metastases and death from prostate cancer in men with conservatively managed, clinically localised cancers (Chodak *et al*, 1994; Albertsen *et al*, 1995, 1998, 2005a; Adolfsson *et al*, 1997; Holmberg *et al*, 2002; Johansson *et al*, 2004; Bill-Axelson *et al*, 2005). Two of these (Albertsen *et al*, 1998; Johansson *et al*, 2004) estimated outcome based on histological tumour grade, but did not include either clinical stage or initial serum PSA level and reviewed Gleason grades. The former (Albertsen *et al*, 1998) reported long-term outcomes for 767 men aged 55–74 years at diagnosis with conservatively treated clinically localised prostate cancer. Men with cancers that have Gleason scores of 2–4, 5, 6, 7, and 8–10 had a 4–7, 6–11, 18–30, 42–70, and 60–87% chance, respectively, of dying from prostate cancer within 15 years of diagnosis, depending on their age at diagnosis. Their revised estimate of 20-year survival (Albertsen *et al*, 2005a) indicated that annual mortality rates remained stable after 15 years from diagnosis. In contrast to these findings, the latter study (Johansson *et al*, 2004) reported an approximately three-fold increase in the rate of progression and prostate cancer-specific mortality rate after 15 years among their small cohort of 223 patients.

The first randomised trial of primary treatment with curative intent for men with localised prostate cancer was reported by Holmberg *et al* (2002) and later by Bill-Axelson *et al* (2005). This trial included 695 men, with clinically localised prostate cancer, randomised to either radical prostatectomy or no initial treatment with systemic treatment deferred until the development of symptomatic progression. The authors found a reduced risk of progression and death from prostate cancer in the radically treated men, but concluded that the disease-specific benefits of radical prostatectomy must be weighed carefully against the potential impact that surgery can have on quality of life. Other studies (Chodak *et al*, 1994; Albertsen *et al*, 1995, 1998, 2005a; Adolfsson *et al*, 1997; Holmberg *et al*, 2002) have concluded that watchful waiting or no initial treatment with treatment delayed until the development of symptomatic metastatic disease remains a viable treatment option.

These studies emphasise the varied natural history of clinically localised prostate cancer, especially for the intermediate risk prostate cancers with Gleason score 6–7.

A basic tenet of an effective screening programme is that the natural history of the disease should be understood well and that early detection can have an important impact on outcome. Neither of these requirements has been satisfied for prostate screening. This study was performed to evaluate the utility of whether other factors such as PSA, clinical stage and extent of disease could provide useful prognostic information in addition to histological grade.

## MATERIALS AND METHODS

### Study population and data collection

This was a population-based study in which potential cases were identified from six cancer registries in Great Britain. Within each region, collaborating hospitals were sought and cases from these hospitals were reviewed. National approval was obtained from the Northern Multi-Research Ethics Committee, followed by local ethics committee approval at each of the collaborating hospital trusts (Appendix A).

Men were included in this study if they were under age 76 years at the date of diagnosis and had clinically localised prostate cancer diagnosed by transurethral resection of the prostate (TURP) or needle biopsy. Diagnosis between 1990 and 1996 (inclusively) and a baseline PSA were required.

Patients treated by radical prostatectomy or radiation therapy within 6 months of diagnosis were excluded. In addition, those with objective evidence of metastatic disease (by bone scan, X-ray, radiograph, CT scan, MRI, bone biopsy, lymph node biopsy, pelvic lymph node dissection) or clinical indications of metastatic disease (including pathologic fracture, soft tissue metastases, spinal compression, or bone pain), or a PSA measurement over 100 ng ml^−1^ at or within 6 months of diagnosis were also excluded. These exclusions were a pragmatic method of focusing the study on patients who were very likely to have truly localised disease at presentation. Men who had hormone therapy prior to diagnostic biopsy were also excluded, because of the influence of hormone treatment on interpreting Gleason grade. We also excluded men who died within 6 months of diagnosis, or had less than 6 months of follow-up.

**Figure 1:**
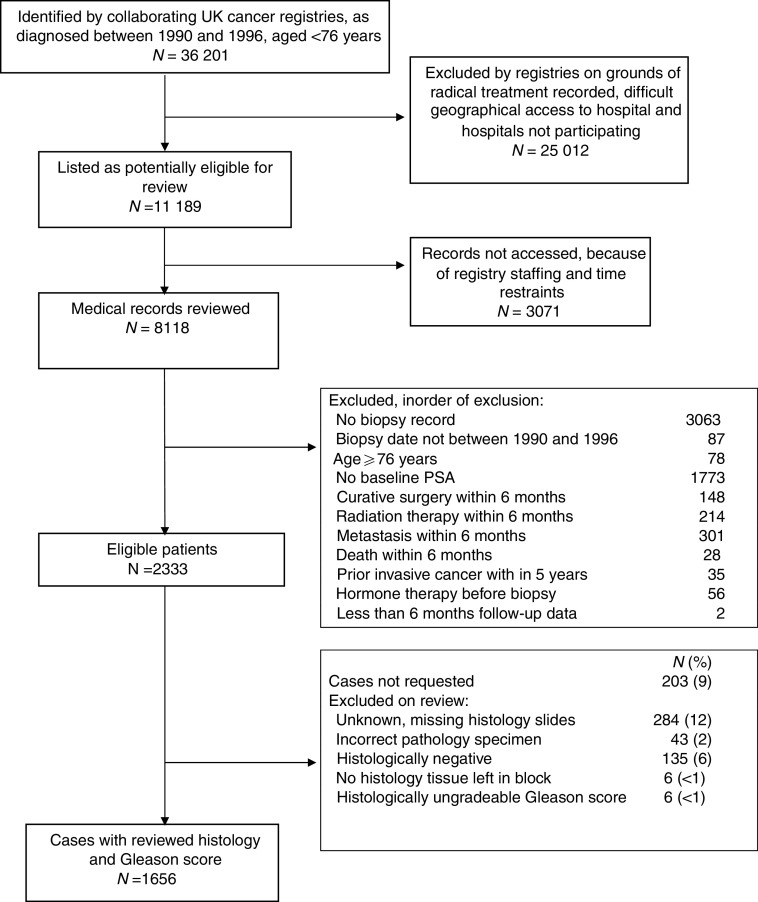
Cohort assembly.

Registry data had limited utility for eliminating ineligible cases, thus a review of hospital records was necessary to establish eligibility. The review process and selection of cases is summarised in Figure 1.

Registry data collection officers and trained medical staff conducted on-site medical record reviews at each of 51 hospital trusts (Appendix A).

Clinical staging was centrally reviewed and, where unstated in the notes, was assigned, where possible, by an urologist based on the reported findings. In approximately 24% of cases, no information was available and in a further 16% of cases, stage could not be assigned. In both circumstances, these cases were designated Tx.

Original histological specimens from the diagnostic procedure (needle biopsy or TURP) were requested, collected, and centrally reviewed by a panel of expert urological pathologists to confirm the diagnosis and, where necessary, to reassign Gleason grades for all the prostate cancers using a conventional interpretation (Deshmukh and Foster, 1997) of the Gleason grading system. Approximately 12% of requested specimens were missing or unidentifiable in hospital pathology databases. A further 17% of cases had no Gleason grade assigned for a variety of reasons (Figure 1). Outcomes were determined through medical records and cancer registry data. In January 2005, the cancer registries were queried to obtain the most up-to-date survival data. Date of last follow-up was different for each cancer registry; the earliest was March 2004 and the latest was January 2005. Where available, death certificates for deceased patients were reviewed to verify cause of death. Deaths were divided into two categories, death from prostate cancer and death from other causes, according to standardised World Health Organisation criteria. Patients still alive at last follow-up were censored at that date.

Disease progression (treatment failure) was defined as clinical, histological, or radiographic evidence of metastatic disease (lymph node, bone, or soft tissue); or institution of additional hormone therapy, radiation therapy, surgery, chemotherapy, or death certified to be from prostate cancer.

### Statistics

The primary end point to this study was time to death from prostate cancer. An initial analysis characterised patient status at different follow-up times (death from prostate cancer, death from other causes, alive with progression, alive without progression) in which censoring was done only if alive and progression free at last follow-up. Subsequent analyses of the main end points were performed by proportional hazards models, censoring at the time of death from other causes, or latest follow-up time. All follow-up times commenced at the point of 6 months following diagnosis. The following variables were recorded: Gleason score, all available PSA values, clinical stage, extent of disease (proportion of TURP chips with disease or linear proportion of needle biopsy containing disease), age at diagnosis, method of diagnosis (TURP or needle biopsy), and initial treatment (no initial treatment or early hormone management). Baseline PSA was defined as the last PSA value within 6 months of diagnosis (including pre-diagnostic values), but before initiation of hormone therapy and at least 3 weeks after any biopsy. Patients for which any of these values greater than 100 ng ml^−1^ were excluded.

Variables were first examined separately and then multivariate models were constructed by a forward stepwise selection method. For the multivariate models, a single linear trend variable across categories was used for assessing the importance of a new variable. If the variable was included, a second variable indicating missing (unassigned) data was added before proceeding to examine further variables. This was done to avoid loss of patients when one variable was missing. A predictor was developed using Gleason score and separate categories of PSA to create prognostic groups. All *P*-values are two-sided and 95% confidence intervals were based on the normal distribution with parameters derived from partial likelihood calculations.

## RESULTS

### Cohort assembly

The process of identification of eligible patients is summarised in Figure 1.

**Figure 2:**
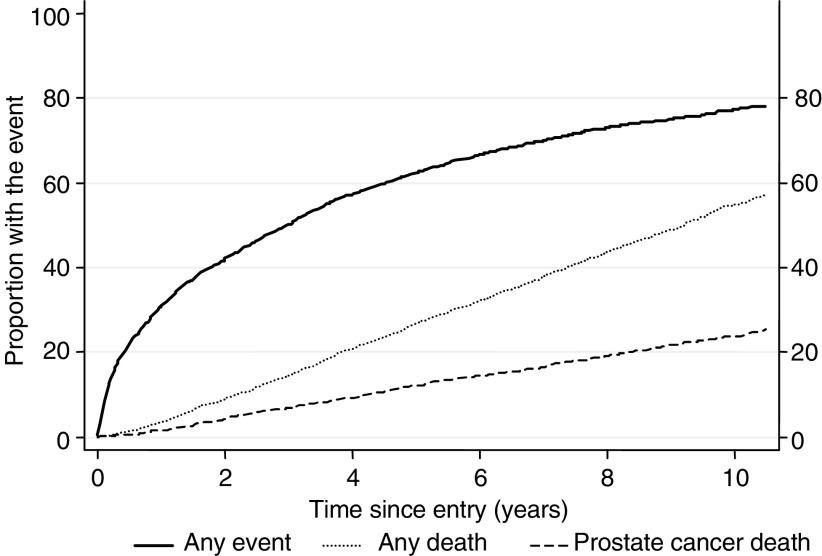
Proportion of patients either dead from prostate cancer, dead from any cause, or dead, progressed or with treatment failure, at different follow-up times.

Of 2333 men eligible for evaluation, 1663 were managed by no initial treatment and 670 were managed by early hormone therapy. Overall, the median age at diagnosis was 70.1 years (range 44–76 years) and the median follow-up was 117 months (range 88–180 months). Most men (80%) were diagnosed after age 65 years. A competing risk analysis showed that after 10 years of follow-up, 55% of the men had died, 24% from prostate cancer and 31% from other causes and only 22% were still alive without progression (Figure 2).

Early hormone therapy treated patients were diagnosed more recently and had a shorter follow-up time, but the age at diagnosis was similar for both cohorts (*P*=0.25). Diagnosis was made by TURP in 1255 men (54%), needle biopsy in 1039 men (45%) and was unspecified in 39 men (1%). Patients treated early by hormone manipulation had a worse prognosis, even after multivariate adjustment. Consequently, results are also given separately for the two methods of initial management.

The distribution of baseline factors and the univariate risk of death from prostate cancer for these factors are shown in Table 1. Separate analyses for patients with early hormone therapy and no initial treatment are given in Appendix A (Table A1).

### Gleason grade

Reviewed Gleason scores were available for 71% of the total cohort, 71% of those treated by no initial treatment, and 72% of those treated by early hormone therapy. Gleason score had the greatest discriminating power, even though this was based on a subset of the cohort (Table 1). A clear gradation was seen across groups with a χ^2^ (trend) of 186, in the total cohort, for prostate cancer death. The predictive power was weaker in patients initially treated by early hormone therapy, but was still stronger than any other variable for this group. Patients, whose histology was not available for review, were similar to the overall group, with a survival curve very similar to those with a Gleason score of 7. Those with a Gleason score of 4 or 5 had a 10-year prostate cancer survival rate of 92% compared to 41% for those with Gleason score 9 or 10. We further subdivided the Gleason score 7 into 3+4 and 4+3, but they behaved similarly (HR 2.17 *vs* 2.51, 10 years survival 73 *vs* 68% respectively).

### Baseline PSA

Baseline PSA values were the second most useful variable and almost as discriminating as Gleason score. The *χ*^2^ (trend) was 153 for prostate cancer death. Again, a clear gradient was seen with survival at 10 years, being 86% for men with PSA values <4 ng ml^−1^, but only 46% for men with values between 50 and 100 ng ml^−1^. Much of the impact of PSA was independent of Gleason score, as can be seen from the multivariate analysis (Table 2a). Low values were less predictive for patients treated by early hormone management.

### Clinical stage and extent of disease

A total of 1387 (60%) patients had sufficient information available to assign a clinical stage. In univariate analysis, a clear but smaller difference than for Gleason score or baseline PSA was seen. Extent of disease proved to be more useful than clinical stage for both cohorts (Table 1) and retained significance in multivariate models.

### Age, method, and year of diagnosis

Age had a clear effect on non-prostate cancer death as expected. The 10-year death rates from other causes were 18, 30, 42, 47% for ages <65, 65–70, 71–73, 74–75 respectively (*χ*^2^ (trend)=104). An effect was also seen for death from prostate cancer. However, no effect was seen on progression rates (data not shown), suggesting this may represent confounding with other established risk factors, or misclassification of cause of death in the elderly.

Method of diagnosis (TURP or needle biopsy) and year of diagnosis had little impact on outcome.

### Multivariate model

A forward stepwise selection multivariate model was developed. The variables were entered in the following order and their relative strengths indicated by the increment in *χ*^2^ (Δ*χ*^2^): Gleason Grade (186.4), PSA (84), age (15.2), percentage cancer in the biopsy (9.8), clinical stage (8.0), and year of diagnosis (2.8). Full details are given in Appendix A (Table A2 (a)). Separate models for no initial treatment and early hormone therapy are provided also in Appendix A (Table A2 (b) and (c)).

The most predictive variable was Gleason score, followed by baseline PSA, age, extent of disease, and clinical stage. Method of initial treatment was also important, even after adjustment for other factors, reflecting an additional selection of poor risk patients among those given early hormone therapy.

A multivariate model for prostate cancer death based only on different levels of Gleason score and baseline PSA is shown in Table 2a and Table 2b.

This was clearly better than the univariate models. Almost all of the information was contained in Gleason score and baseline PSA. With these two variables we were able to identify 17% of the cohort for which the Gleason score was⩽5 and PSA<25 ng ml^−1^ or the Gleason score was 6 and PSA⩽4 ng ml^−1^ where prostate cancer mortality at 10 years was less than 10% and 33% of the cohort with Gleason score 7 and PSA>25 ng ml^−1^, or Gleason score 8 and PSA>10 ng ml^−1^, or Gleason score⩾9 where prostate cancer mortality at 10 years was greater than 30%. This last group could be further split into a very poor prognostic group (Gleason score⩾9 and PSA>25 ng ml^−1^) comprising 5% of men with a 10-year prostate cancer mortality greater than 75% (Table 2b).

**Figure 3:**
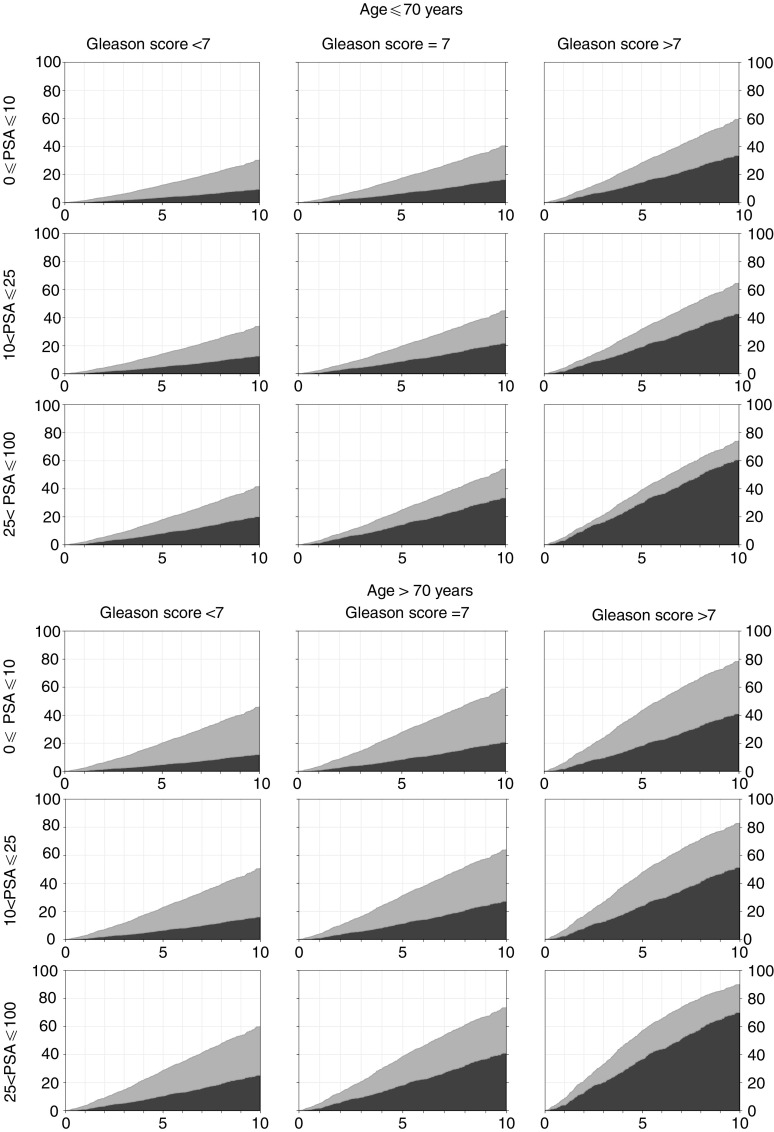
Proportion of patients dead from prostate cancer (dark grey), dead from other causes (light grey), at different follow-up times up to 10 years, according to baseline PSA and Gleason score, separately for patients aged 70 years or less at diagnosis and patients aged more than 70 years at diagnosis.

The relative importance of death from prostate cancer *vs* other causes over the first 10 years of follow-up for different prognostic groups is shown (Figure 3) separately for men aged 70 years or less and men aged more than 70 years, at diagnosis.

## DISCUSSION

Prostate cancer is currently the second leading cause of cancer death among men in both the UK and USA. However, approximately half the men diagnosed with this disease do not die from it, even in the absence of radical treatment (Satariano *et al*, 1998). This makes counselling patients about management difficult. Recent results from the Scandinavian Prostate Cancer Study Group (Bill-Axelson *et al*, 2005) demonstrate a reduction in metastatic disease and prostate cancer-specific mortality for radical prostatectomy compared to watchful waiting, but the gains are small and it is not clear which men will benefit in this. Our data incorporate informative prognostic parameters not analysed in previous studies on conservatively treated men. In agreement with Albertsen *et al* (1995) and Johansson *et al* (2004), we found Gleason score to be an important determinant of cancer-specific mortality. In addition, we found baseline PSA level and to a lesser extent clinical stage and extent of disease added further predictive value. Importantly, the information contained in PSA levels was largely independent of that available from Gleason score and *vice versa*, so that using both variables produced a simple classification into three groups with very different outcomes.

Compared to previous studies, there are several reasons why our findings are more applicable to a man diagnosed with localised prostate cancer in the modern era. Firstly, the incorporation of PSA augments the prognostic stratification by histological grading alone. Secondly, the Gleason scoring in our study more accurately reflects current pathological grading methods. Multiple investigators have documented an upward shift in Gleason score over time (Kondylis *et al*, 2003; Albertsen *et al*, 2005b) – not representative of a change in biology, but of pathologists today being more likely to assign higher Gleason scores. Albertsen *et al* (2005a, 2005b) assigned Gleason grades in 1990–1992 and 33% of patients had a Gleason score of 5 or less compared to our study where only 3% patients had this classification. Thirdly, in Albertsen's cohort, 24% of patients had a Charlson Index (Charlson *et al*, 1987) of two or greater, considered to be ‘significant co morbidity’. Consequently, the cohort represents probably a high proportion of patients not medically suitable, or patients deciding not to opt for primary curative therapy. Since conservative management was used for a much broader group of patients in the United Kingdom, our population-based cohort may be a more representative one for men faced with the choice of conservative *vs* curative-intent therapy. While 54% of our patients were diagnosed by TURP, which is much higher than would be found in a contemporary series, this did not have an impact on outcomes, PSA levels were on average also higher than for current series, but there were sufficient numbers of patients in all groups to obtain reliable prognostic information across the full spectrum of values. Lastly, our cohort is more than three times the size of previous studies, providing more accurate estimates of risk and greater statistical power.

Kattan *et al* (1998) found clinical stage, PSA, and Gleason grade to be parameters predictive of PSA recurrence for men treated by radical prostatectomy. In our study, clinical stage provided only a small additional amount of information on prostate cancer death. However, our information on clinical stage was unavailable for 40% of the cohort, and were recorded, was limited and based on a retrospective review of clinical notes from a variety of institutions. It is possible that carefully collected and complete prospective information would prove to be more prognostic. This is in contrast to the data on PSA that was 100% complete and centrally reviewed Gleason Grade, which was available for 71% of the cohort. Age was a strong predictor of death from causes other than prostate cancer, and provided some additional information on prostate cancer mortality. However, it did not predict progression, and its effect on prostate cancer mortality may reflect misclassification of cause of death in these elderly men.

Our analyses identified 33% of men with poor 10-year cancer-specific mortality (>30%), where no initial treatment is not a good option, especially when the risk of death from other causes is low. We also identified a group comprising 17% of men where the 10-year cancer-specific survival was very good (>90%). Long follow-up will be needed to see if these men remain at low risk of disease-related mortality, since it is not clear if mortality rate trends will plateau or continue to increase. In the Swedish (Johansson *et al*, 2004) cohort, cancer-specific mortality rates increased during years 15–20 following diagnosis relative to the initial 15 years, whereas Albertsen *et al* (2005a) found annual cancer-specific mortality rates to be unchanged after 15 years.

In our study, approximately 50% of patients have what may be considered an intermediate prognosis (10–30% 10-year cancer-specific mortality), where better markers of disease progression are needed. This applies especially to the 11% of men with Gleason score 6 and PSA level 4–10 ng ml^−1^, many of whom undoubtedly have a very good prognosis. Currently, we are collecting all available tumour blocks from the cohort to construct tissue micro-arrays. It is hoped that identification of new markers with altered DNA or protein expression in prostate cancer will help to highlight disease destined to be clinically relevant, especially for this intermediate group.

In conclusion, we have confirmed Gleason score as an important prognostic factor for men with conservatively treated localised prostate cancer and are the first to include PSA at diagnosis into the prognostic model for these patients. While providing valuable information for men considering or choosing conservative therapy, our study has also emphasised the urgent need to identify better markers of tumour behaviour to assist in formulating appropriate management of individual men with prostate cancer.

## Figures and Tables

**Table 1:** Univariate analysis of factors influencing death from prostate cancer in men with conservatively managed clinically localised prostate cancer (total eligible cohort=2333)

**Variable**	***N* (%)**	**Hazard ratio (95% CI)**	**Prostate cancer death at 10 years (%)**
*Gleason score* a			
⩽5	61 (3)	0.59 (0.2–1.6)	8
6	681 (29)	1^b^	13
7	500 (21)	2.30 (1.7–3.1)	29
8	250 (11)	3.89 (2.8–5.3)	41
9 or 10	164 (7)	8.22 (6.0–11.3)	61
Unassigned^c^	677 (29)	2.55 (1.9–3.4)	31
		*χ*^2^ (trend)=186	
			
*Serum PSA (ng ml^−1^)*			
⩽4	546 (23)	1^b^	14
>4–10	502 (21)	1.34 (1–1.9)	19
>10–25	607 (26)	2.05 (1.5–2.8)	28
>25–50	400 (17)	3.19 (2.4–4.3)	38
>50–100	278(13)	5.06 (3.7–6.8)	54
		*χ*^2^ (trend)=153	
			
*Clinical stage*			
T1	506 (22)	0.4 (0.3–0.5)	13
T2	612 (26)	1^b^	30
T3	269 (12)	1.67 (1.3–2.2)	40
Tx^c^	946 (40)	0.94 (0.8–1.2)	30
		*χ*^2^ (trend)=32	
			
*Cancer in biopsy (%)*			
⩽6	341 (15)	1^b^	11
>6–20	386 (17)	1.47 (0.9–2.3)	16
>20–40	278 (12)	2.89 (1.9–4.4)	32
>40–75	336 (14)	3.56 (2.4–5.3)	34
>75–100	313 (13)	4.90 (3.3–7.2)	43
Unspecified^c^	679 (29)	2.91 (2–4.2)	30
		*χ*^2^ (trend)=105	
			
*Age (years)*			
⩽65	474(20)	0.72 (0.6–0.9)	21
>65–70	665 (29)	1^b^	25
>70–73	594 (25)	1.1 (0.9–1.4)	28
>73–76	600 (26)	1.41 (1.1–1.8)	35
		*χ*^2^ (trend)=25	
			
*Year of diagnosis*			
1990	54 (2)	1.14 (0.7–2)	29
1991	109 (5)	1.35 (0.9–2)	32
1992	225 (10)	1.44 (1–2.0)	33
1993	346 (15)	1.29 (1–1.7)	32
1994	471 (20)	1.02 (0.8–1.4)	24
1995	551 (24)	1.05 (0.8–1.4)	23
1996	577 (2)	1^b^	(not yet achieved)
		*χ*^2^ (trend)=5.4	
			
*Method of diagnosis*			
Needle Biopsy	1039 (45)	1^b^	31
TURP	1255 (54)	0.81 (0.7–1)	25
Unspecified^c^	39 (1)	0.95 (0.5–1.8)	27
		*χ*^2^=4.7	
			
*Baseline hormones*			
No	1663 (71)	1^b^	20
Yes	670 (29)	2.6 (2.2–3.1)	46
		*χ^2^=113*	

CI denotes confidence interval.

a Score assigned during histopathological review.

b Reference category.

c These cases were excluded from the trend analysis.

PSA denotes prostate specific antigen.

TURP denotes trans urethral resection of the prostate.

**Table 2a:** Hazard ratios for multivariate model for prostate cancer death based on Gleason score and PSA, in patients having both variables available

	**Prostate cancer death**		
**Variable**	**Total eligible (*N*=1656)**	**No initial treatment (*N*=1176)**	**Initial hormones (*N*=480)**
*Gleason score* a			
⩽5	0.67	0.63	1.50
6	1^b^	1^b^	1^b^
7 (3+4, 4+3)	1.77	1.88	1.13
8 (3+5, 4+4, 5+3)	3.00	3.26	1.79
9 or 10	5.96	6.77	3.56
			
*Serum PSA (ng ml^−1^)*			
⩽4	1^b^	1^b^	1^b^
>4–10	1.06	0.81	1.05
>10–25	1.42	1.60	0.65
>25–50	2.23	1.67	1.73
>50–100	2.62	1.92	1.67

a Score assigned during histopathological review.

b Reference category.

**Table 2b:**
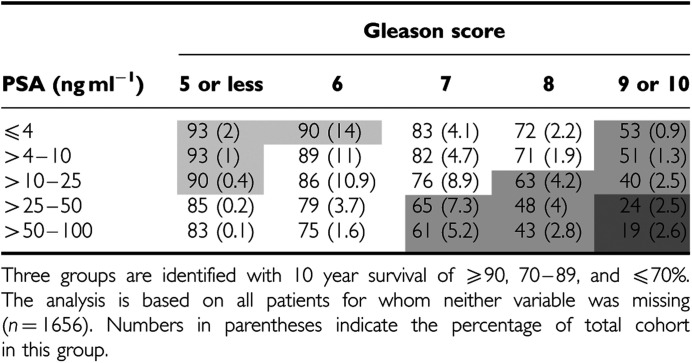
Predicted prostate cancer specific survival at 10 years based on baseline PSA and Gleason score, in men with clinically localised disease

**Table A1:** Univariate analysis of factors influencing death from prostate cancer in men with clinically localised prostate cancer, (a) and given no initial treatment: *N*=1663 and (b) initially treated with hormones: *N*=670

**Variable**	***N* (%)**	**Hazard ratio (95% CI)**	**Prostate cancer death at 10 years (%)**
(a)			
*Gleason score* a			
⩽5	57 (3)	0.59 (0.2–1.9)	6
6	589 (35)	1^b^	10
7	313 (19)	2.24 (1.5–3.3)	23
8	136 (8)	3.84 (2.5–5.9)	38
9 or 10	81 (5)	8.64 (5.7–13.2)	53
Unassigned^c^	487(29)	2.39 (1.7–3.4)	24
		*χ*^2^ (trend)=101	
			
*Serum PSA (ng ml*^−*1*^)			
⩽4	513 (31)	1^b^	12
>4–10	416 (25)	1.14 (0.8–1.7)	15
>10–25	432 (26)	2.13 (1.5–3)	27
>25–50	212 (13)	2.64 (1.8–3.9)	30
>50–100	90 (5)	4.1 (2.6–6.3)	41
		*χ* ^2^(trend)=56	
			
*Clinical stage*			
T1	435 (26)	0.42 (0.3–0.6)	10
T2	395 (24)	1^b^	22
T3	126 (8)	1.69 (1.1–2.5)	33
Tx^c^	707 (43)	1.02 (0.8–1.4)	24
		*χ* ^2^(trend)=21	

*Cancer in biopsy* (%)			
⩽6	315 (19)	1^b^	10
>6–20	308 (19)	1.16 (0.7–2)	10
>20–40	196 (12)	2.75 (1.7–4.5)	27
>40–75	202 (12)	3.73 (2.3–6)	29
>75–100	156 (9)	4.3 (2.6–7)	33
Unspecified^c^	486 (29)	2.58 (1.7–4)	24
		*χ*^2^=59	
			
*Age (years)*			
⩽65	357 (21)	0.7 (0.5–1)	14
>65–70	466 (28)	1^b^	18
>70–73	417 (25)	1.23 (0.9–1.7)	22
>73–76	423 (25)	1.6 (1.2–2.2)	28
		*χ*^2^ (trend)=23	
			
*Year of diagnosis*			
1990	46 (3)	1.68 (0.9–3.2)	29
1991	86 (5)	1.73 (1.1–2.8)	30
1992	168 (10)	1.71 (1.1–2.6)	25
1993	266 (16)	1.27 (0.8–1.9)	22
1994	344 (21)	1.1 (0.7–1.7)	18
1995	370 (22)	0.97 (0.6–1.5)	15
1996	383 (23)	1^b^	(not yet achieved)
		*χ*^2^ (trend)=11	
			
*Method of diagnosis*			
Needle biopsy	611 (37)	1^b^	23
TURP	1023 (61)	0.83 (0.7–1.1)	19
Unspecified^c^	29 (2)	0.9 (0.4–2.2)	19
		*χ*^2^=1.9	
			
(b)			
*Gleason score reviewed* a			
⩽5	4 (1)	1.30 (0.2–9.7)	—
6	92 (14)	1^b^	39
7	187 (28)	1.28 (0.8–2.1)	40
8	114 (17)	1.92 (1.1–3.2)	46
9 or 10	83 (12)	3.82 (2.3–6.4)	68
Unassigned^c^	190 (28)	1.78 (1.1–2.9)	48
		*χ*^2^ (trend)=33	
			
*Serum PSA (ng ml*^−*1*^)			
⩽4	33 (5)	1^b^	36
>4–10	86 (13)	1.03 (0.5–2.2)	40
>10–25	175 (26)	0.79 (0.4–1.6)	32
>25–50	188 (28)	1.42 (0.7–2.8)	48
>50–100	188 (28)	1.95(1–3.9)	62
		*χ*^2^ (trend)=20	
			
*Clinical stage*			
T1	71 (11)	0.7 (0.4–1.2)	34
T2	217 (32)	1^b^	44
T3	143 (21)	1.32 (0.9–1.9)	46
Tx^c^	239 (36)	1.04 (0.8–1.4)	51
		*χ*^2^(trend)=2	
			
*Cancer in biopsy* (%)			
⩽6	26 (4)	1^b^	33
>6–20	78 (12)	1.13 (0.5–2.6)	40
>20–40	82 (12)	1.41 (0.6–3.2)	44
>40–75	134 (20)	1.28 (0.6–2.9)	40
>75–100	157 (23)	1.87 (0.9–4.1)	53
Unspecified^c^	193 (29)	1.56 (0.7–3.4)	48
		*χ*^2^=5.8	
			
*Age (years)*			
⩽65	117 (18)	0.88 (0.6–1.3)	42
>65–70	199 (30)	1^b^	42
>70–73	177 (26)	1.0 (0.7–1.4)	46
>73–76	177 (26)	1.24 (0.9–1.7)	54
		*χ*^2^(trend)=2.8	
			
*Year of diagnosis*			
1990	8 (1)	0.65 (0.2–2.1)	25
1991	23 (3)	1.18 (0.6–2.3)	40
1992	57 (9)	1.39 (0.9–2.3)	53
1993	80 (12)	1.75 (1.2–2.6)	63
1994	127 (19)	1.08 (0.7–1.6)	41
1995	181 (27)	1.16 (0.8–1.7)	40
1996	194 (29)	1^b^	(not yet achieved)
		*χ*^2^(trend)=1.7	
			
*Method of diagnosis*			
Needle biopsy	428 (64)	1^b^	42
TURP	232 (35)	1.37 (1.1–1.8)	52
Unspecified^c^	10 (1)	1.26 (0.5–3.1)	50
		*χ*^2^=4.8	

CI denotes confidence interval.

a Score assigned during histopathological review.

b Reference category.

c These cases were excluded from the trend analysis._[2]_PSA denotes prostate-specific antigen.

TURP denotes trans urethral resection of the prostate.

**Table A2:** Multivariate model for prostate cancer death based on Gleason Grade (G), PSA (P), age (A), percentage cancer in biopsy(C), clinical stage (T), year (Y) and (a) initial hormone treatment (H), (b) In patients with no initial treatment, and (c) Patients with initial treatment by early hormone therapy

**Variables**	**df**	***N***	****χ** ^2^**	**Δ**χ**^2^ (1 df)**
(a)				
*Total eligible cohort*				
**G**	1	1656	186.42	186.4
G Gm	2	2333	188.08	
G Gm **P**	3	2333	272.10	84.0
G Gm P **A**	4	2333	287.34	15.2
G Gm P A (restricted – if C not missing)	4	1654	239.45	
G Gm P A **C** (restricted – if C not missing)	5	1654	249.20	9.8
G Gm P A C Cm (restricted – if T not missing)	6	1751	231.37	
G Gm P A C Cm **T** (restricted – if T not missing)	7	1751	239.34	8.0
G Gm P C Cm A T Tm	8	2333	305.12	
G Gm P C Cm A T Tm **Y**	9	2333	307.96	2.8
G Gm P C Cm A T Tm Y **H**	10	2333	328.65	20.7
				
(b)				
*No initial treatment cohort*				
**G**	1	1176	100.53	100.5
G Gm	2	1663	104.07	
G Gm **P**	3	1663	130.72	26.7
G Gm P **A**	4	1663	144.81	14.1
G Gm P A (restricted – if C not missing)	4	1177	117.64	
G Gm P A **C** (restricted – if C not missing)	5	1177	126.47	8.8
G Gm P A C Cm (restricted – if T not missing)	6	1219	111.29	
G Gm P A C Cm **T** (restricted – if T not missing)	7	1219	119.31	8.0
G Gm P C Cm A T Tm	8	1663	162.37	
G Gm P C Cm A T Tm **Y**	9	1663	166.25	3.9
				
(c)				
*Initial treatment cohort*				
**G**	1	480	33.39	33.4
G Gm	2	670	33.14	
G Gm **P**	3	670	50.67	17.5
G Gm P **A**	4	670	54.28	3.6
G Gm P A (restricted – if C not missing)	4	477	52.66	
G Gm P A **C** (restricted – if C not missing)	5	477	52.85	0.2
G Gm P A C Cm (restricted – if T not missing)	6	532	46.11	
G Gm P A C Cm **T** (restricted – if T not missing)	7	532	46.73	0.6
G Gm P C Cm A T Tm	8	670	55.34	
G Gm P C Cm A T Tm **Y**	9	670	56.45	1.1

## Appendix A

Members of the Transatlantic Prostate Group included listed authors and investigators designated by an asterisk.

Investigators in participating regional cancer registries and hospital trusts are listed below:

Thames Cancer Registry: Henrik Møller^*^, Shirley Bell (deceased), K Linklater, J Ottey V Fisher; Ashford & St Peter's, M Hall, N Harvey Hills; Barnet & Chase Farm, H Reid; Brighton and Sussex, N Kirkham, P Thomas; Bromley, D Nurse; Dartford & Gravesham, I Dickinson, P Thebe; East & North Hertfordshire, D Hanbury, M Ali-Izzi; Eastbourne, C Moffatt; Epsom & St Helier, M Bailey, L Temple; Essex Rivers Healthcare, W Aung, C. Booth; Frimley Park, B Montgomery, P Denham; Greenwich Healthcare, N Cetti, P Pinto; Guy's & St Thomas's, A Chandra, T O’Brien; Hammersmith Hospitals, N Livni; Havering Hospitals, I Saeed; Hillingdon, F Barker, T Beaven; King's Healthcare, G Muir, Z Khan; Kingston, C Jameson; Lewisham, A Giles; Mayday Healthcare, N Arsanious, A Arnaout; The Medway, E Boye; Mid Essex Hospitals; Mid Kent, M Boyle; North West London Hospitals, M Jarmulowicz; Royal Free Hampstead, RJ Morgan, A Bates; St Bartholomew's and The Royal London Hospitals, F Chinegwundoh, RTD Oliver, D Berney; Royal Surrey County, S De Sanctis; Southend, M Chappell; St George's, London, R Kirby, C Corbishley; St Mary's, London, A Patel, M Walker; West Hertfordshire, J Crisp, W Riddle; Worthing & Southlands Hospitals, J Grant.

Northern & Yorkshire Cancer Registry & Information Service: David Forman^*^, C Storer, C Bennett, C Spink; Airedale, I Appleyard, J O’Dowd; Hull & East Yorkshire, J Hetherington, A MacDonald; The Leeds Teaching Hospitals, P Whelan, P Quirke, P Harnden.

Oxford Cancer Intelligence Unit: Monica Roche^*^, Sandra Edwards, S Bose, P Hall; Heatherwood & Wexham Park, M Ali, O Karim; Milton Keynes, E Walker, S Jalloh; Northampton, M Miller, A Molyneux; Oxford Radcliffe, S Brewster, D Davies; Royal Berkshire & Battle, P Malone, C McCormick; Stoke Mandeville, J Greenland, A Padel

Welsh Cancer Intelligence & Surveillance Unit: John Steward^*^, Shelagh Reynolds, Lynda Roberts, Judith Adams; Ceredigion and Mid Wales, J Edwards, CGB Simpson; Conwy & Denbighshire, A Dalton, V Srinivasan; NE Wales, A De Bolla, C Burdge; Gwent Healthcare, W Bowsher, M Rashid; Swansea, M Lucas, C O’Brien; Cardiff & Vale, M Varma.

Scottish Cancer Registry: David Brewster^*^; The Lothian University Hospitals, J Royle, K Grigor; North Glasgow University Hospitals, D Kirk, A Milano, R Reid.

Merseyside & Cheshire Cancer Registry: Lyn Williams^*^, R Iddenden; Royal Liverpool University Hospital, CS Foster, P Cornford.

Memorial Sloan Kettering Cancer Center: H Lilja^*^, S Eggener^*^.
